# Isotopic Composition of C, N, and S as an Indicator of Endometrial Cancer

**DOI:** 10.3390/cancers16183169

**Published:** 2024-09-16

**Authors:** Tomasz Zuzak, Anna Bogaczyk, Agnieszka Anna Krata, Rafał Kamiński, Piotr Paneth, Tomasz Kluz

**Affiliations:** 1 Department of Gynecology, Gynecology Oncology and Obstetrics, Fryderyk Chopin University Hospital, Szopena 2, 35-055 Rzeszow, Poland; tomasz.zuzak@gmail.com (T.Z.); jtkluz@interia.pl (T.K.); 2 Institute of Applied Radiation Chemistry, Lodz University of Technology, Zeromskiego 116, 90-924 Lodz, Poland; agnieszka.krata@p.lodz.pl (A.A.K.); rafal.kaminski@p.lodz.pl (R.K.); 3 Institute of Medical Sciences, Medical College of Rzeszow University, 35-959 Rzeszow, Poland

**Keywords:** endometrial cancer, isotopic fractionation, biomarkers, isotope ratio mass spectrometry

## Abstract

**Simple Summary:**

In this contribution, we show that, at a natural abundance, the nitrogen, carbon, and sulfur isotopic compositions of uterus cancerous and healthy tissues are different due to various metabolic pathways. These differences indicate that the isotopic composition might be a potential diagnostic and prognostic biomarker. We have tried to correlate the isotopic composition of tissues with that of serum, which would give us access to noninvasive biomarkers. In this respect, the obtained bulk isotopic compositions do not show any systematic correlation between the cancerous tissue and serum. Their application as biomarkers would probably require a position-specific isotopic analysis.

**Abstract:**

Objectives: The metabolic pathway of cancerous tissue differs from healthy tissue, leading to the unique isotopic composition of stable isotopes at their natural abundance. We have studied if these changes can be developed into diagnostic or prognostic tools in the case of endometrial cancer. Methods: Measurements of stable isotope ratios were performed using isotope ratio mass spectrometry for nitrogen, carbon, and sulfur isotopic assessment. Uterine tissue and serum samples were collected from patients and the control group. Results: At a natural abundance, the isotopic compositions of all three of the studied elements of uterus cancerous and healthy tissues are different. However, no correlation of the isotopic composition of the tissues with that of serum was found. Conclusions: Differences in the isotopic composition of the tissues might be a potential prognostic tool. However, the lack of a correlation between the differences in the isotopic composition of the tissues and serum seems to exclude their application as diagnostic biomarkers, which, however, might be possible if a position-specific isotopic analysis is performed.

## 1. Introduction

In the coming decades, cancers are expected to become the leading cause of mortality [1]. The early detection of cancer can significantly increase the chances of a cure [2,3], making the development of effective diagnostic tools and prognostic methods crucial [4,5,6,7]. Stable isotopes at their natural abundance have recently emerged as a promising approach in this regard. The metabolic pathway of cancerous tissue differs from healthy tissue, leading to a unique isotopic composition. High-precision analytical tools such as multi-collector inductively coupled plasma mass spectrometry (MC-ICP-MS) and isotope ratio mass spectrometry (IRMS, usually connected online with an elemental analyzer, the EA-IRMS) have been developed to detect these small differences. Emerging techniques, like clamped isotopes and orbitrap mass spectrometry, have not, to the best of our knowledge, been used to study the isotopic composition of cancerous samples thus far.

The early results of the applications of MC-ICP-MS were summed up in a review in 2016 [8]. The use of the isotopic analysis of zinc (δ66Zn) is analytically challenging due to contaminations [9], while the isotopic composition of iron (δ56Fe) does not differ significantly between cancerous and healthy tissues. On the other hand, the measurements of the isotopic composition of copper (δ65Cu) have been extensively explored. It was concluded that the isotopic composition of copper could be indicative of the survival of patients with hepatocellular carcinoma [10], breast cancer [11] colorectal cancer [11], liver cancer [12], ovarian cancer [13], and bladder cancer [14]. Finding a correlation between the isotopic composition of the cancerous tissue and the serum would allow us to establish a new, noninvasive biomarker, but the problem is subject to debate; while some recent results indicate that such a correlation exists [14], others indicate that the copper isotopic ratio in serum is not related to the tumor, but rather to the condition of an organ [15].

IRMS is mostly dedicated to measuring the isotopic ratios of light elements, predominantly of carbon (δ13C) and nitrogen (δ14N). These isotopic compositions have also been tested for potential applications in cancer studies, and a review covering results obtained with both techniques has been published recently [16]. Among the latest reports, noticeable changes in the isotopic composition of nitrogen between the cancerous and healthy tissues have been shown by Straub and collaborators on mice [17] and human [18] samples, and explained in terms of the nitrogen metabolism. Similar nitrogen fractionation has been found in our work on uterine (reported herein) and bladder cancers [19]. The latter communication also brings an interesting result of the prognostic value of the carbon isotopic composition. The problem with using δ13C as a prognostic or diagnostic tool is, however, the large dilution of the carbon isotopic fractionation connected with a specific site by a large number of non-fractionating (or differently fractionating) carbon atoms, since EA-IRMS measurements are usually carried out for a carbon isotopic analysis on carbon dioxide that originates from burning a tissue sample, and thus averaging the isotopic composition.

Potentially interesting isotopic compositions of oxygen (δ18O) and hydrogen (δ2H) face experimental problems. In the first case, the analysis requires pyrolysis and is prone to contamination. The isotope effects of hydrogen, on the other hand, are very large and their interpretation in terms of an isotopic fractionation of a given site would probably not be very reliable [20]. Sulfur poses a special case, as its isotopic composition can be measured with both techniques and its metabolism (at least in plants) has been shown to exhibit large variations [21]. There are, however, only a few reports where the sulfur isotopic composition has been used in studies of tumors. It has been found that the blood of patients with hepatocellular carcinoma is enriched in the light isotope (a smaller δ34S than the control tissue) [10]. However, in the review on the medical applications of copper, zinc, and sulfur isotope effects, it has been concluded that, except for the studies mentioned above, there is no apparent difference between the sulfur isotopic composition in serum in the cases of patients with colon, breast, and liver cancers and the control groups (although the δ34S spread in the control group was smaller) [8].

In this contribution, we report on studies on tissues and serum for the δ13C, δ15N, and δ34S of patients with uterine cancer, which shed some new light on the isotopic composition variations in carbon and sulfur.

## 2. Materials and Methods

Measurements were performed on bulk tissue and plasma samples taken from patients prior or during surgeries. No attempts have been made to separate their components; thus, no specific characterization of individual compounds (including HPLC or NMR) was possible.

**Figure 1:**
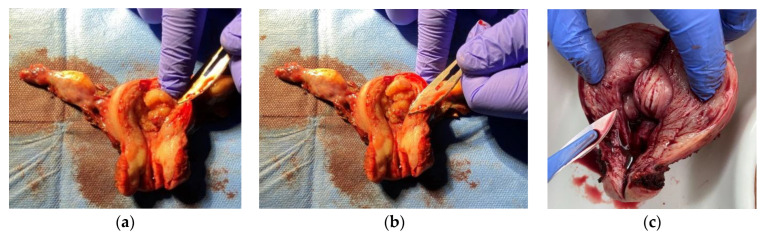
Sites of sample collections: (**a**) from the cancerous tumor, (**b**) from the tissue surrounding the cancerous tumor, (**c**) and from the endometrium.

Thirty-two patients with an average age of 66.9 years (min. 46 and max. 86) with a diagnosis of endometrial cancer were enrolled in the studies reported herein. The histopathological evaluation determined the histological grade G of endometrial cancer (Grade). The study group showed 15 (46.9%) patients with a diagnosis of G1 (a low grade of histological malignancy), 12 (37.5%) patients with a diagnosis of G2 (an intermediate grade of malignancy), and 5 (15.6%) patients with a diagnosis of G3 (a high grade of malignancy). In each case, three tissue blocks measuring 3 × 5 mm directly from the cancerous tumor (Figure 1a), from the area up to 1 cm from the tumor (Figure 1b), and the area most distant from the lesion (Figure 1c), were taken.

All of the patients gave their written consent to the use of tissues for the research: Consent of the Bioethics Committee of the District Medical Chamber of 21 May 2020, resolution no. 54/B/2020.

The whole peripheral blood was collected in both groups each time about 2 h before surgery. This was to avoid interference in the results by the action of infusion fluids necessary for the proper preoperative preparation of the patient. The material was immediately transported to the hospital laboratory, where the peripheral blood serum was obtained by centrifugation and pipetting. The serum was then placed in Eppendorf tubes and frozen at −76 °C until delivered for measurements.

The control group included 31 patients with a mean age of 48.8 years (min. 36 and max. 64). The predominant diagnosis in the control group was uterine myomas (28; 90.3%), followed by increased cervical dysplasia (2; 6.5%) and adenomyosis (1; 3.2). The material for the study was a tissue block of endometrium measuring 3 × 5 mm taken under macroscopic control from a site distant from the uterine lesions, i.e., usually myomas.

In both the control and cancer groups, the collected material was immediately placed in sterile containers without buffer, labeled, and frozen at −76 °C until the delivery for the measurements.

The measurements of the stable isotope ratios were performed with the use of a Sercon HS2022 Continuous Flow Isotope Ratio Mass Spectrometer (IRMS, Sercon Limited, Crewe, UK) connected to a Sercon SL elemental analyzer for simultaneous nitrogen–carbon–sulfur (NCS) assessment. The uterine tissue and serum samples collected from the patients were stored frozen at −70 °C. The samples were carefully prepared before the IRMS analysis. The procedure for the uterine tissue samples included freeze-drying lyophilization for 5 days followed by cutting the samples into small pieces, weighing the samples on an analytical balance (precision ± 0.01 mg, Denver Instrument, Göttingen, Germany), and placing the samples in tin capsules (cylinder, ultra-clean pressed, 8 × 5 mm, Sercon, Cheshire, UK). In the initial series, samples of 1 to 3 mg were used, while, in the second series, masses were increased to about 6 mg. In this series, vanadium pentoxide (Sercon) as a combustion catalyst for sulfur was added. In the case of preparing the serum samples, we developed a home-made vacuum-dried apparatus, and the following procedure was applied: empty tin capsules (cylinder, smooth wall, 7 × 3.5 mm, OEA Labs, Exeter, UK) were weighed and 60 μL of blood plasma was pipetted inside of them. Capsules were dried in a rotator at a pressure below 0.4 mbar until the constant weight had been reached (approximately 105 min.). Again, vanadium pentoxide was added to each sample in the second series. All of the samples were prepared in duplicate and stored at room temperature. The home working standard, thiobarbituric acid, purchased from Sigma-Aldrich (Steinheim, Germany), was used, and the results are reported in δ-notation versus the following primary standards: N2 in air, PeeDee Belemnite (PDB), and Canyon Diablo Troilite (CDT) for δ15N, δ13C, and δ34S, respectively, where δX = (Rsample/Rstandard − 1) × 1000. δX represents ratios of N (15N/14N), C (13C/12C), and S (34S/32S) isotopes expressed in per mil (‰).

## 3. Results

The studies were performed in two parts. The first part was devoted to establishing the protocol of the procedures, both on the surgical and analytical sides. The second part was dedicated to obtaining accurate results. Both parts contained the following two series: K, with control samples, and B, with cancerous material. Each series consisted of samples of serum and the following three samples of tissues: the tumor, the edge of the tumor region, and from a healthy part remote from the tumor by about 5 cm. The second part was optimized for the measurements of the sulfur isotopic composition, which was not measured in the first part of the study. Since the tumor edges were not always clear, we decided not to use these samples in the analysis. Consecutive numbers represent different patients. In the control group, the majority of the tissue was from the uterine fibroid, although, in the first part, the tissue was from an ovarian cyst, and the healthy tissue (from the preventive surgery) was used.

**Figure 2:**
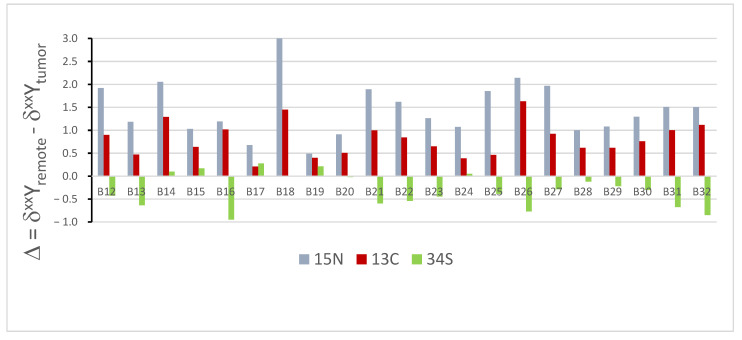
Differences between the isotopic composition of healthy and cancerous tissues.

Figure 2 summarizes the differences between the isotopic composition of the cancerous and healthy tissues for all three of the elements studied, which is expressed in per mil units as

Δ = δ^xx^Y_remote_ − δ^xx^Y_tumor_

where xx stands for the mass of the heavier isotope and Y stands for the element, each for the samples of the isotopic composition of all three of the elements studied. Note the smaller dispersion of Δ oϕ sulfur compared to nitrogen and carbon, and its opposite direction, in most of the samples.

**Figure 3:**
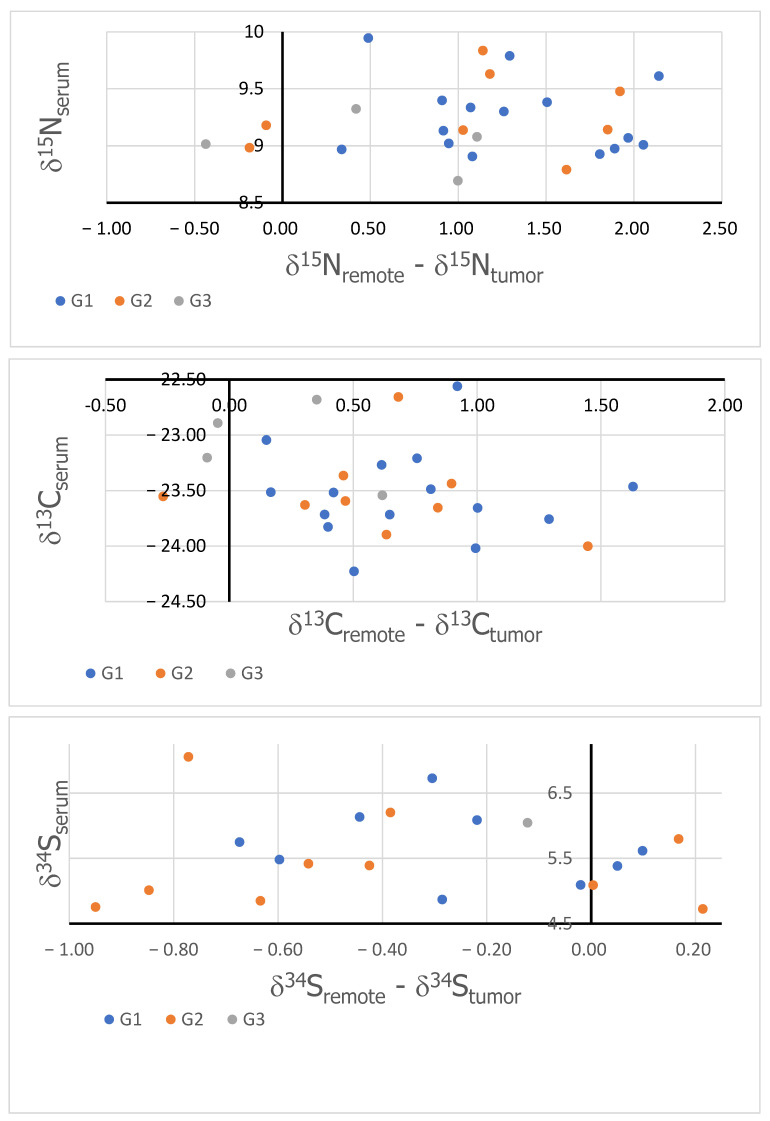
Isotopic compositions of serum in the function of the differences between healthy and cancerous tissues of nitrogen (**top**), carbon (**middle**), and sulfur (**bottom**).

Figure 3 shows the δ values of nitrogen (top), carbon (middle), and sulfur (bottom) measured in serum as the function of the corresponding Δ. The colors mark the different gradings (blue points indicate G1, orange points indicate G2, and green points indicate G3).

## 4. Discussion

Malignant tumor cells, including endometrial cancer, are characterized by significantly different metabolic pathways compared to healthy tissues. This is due to several factors that include genetic, epigenetic, and microenvironmental changes. The so-called Warburg effect is among the most widely discussed in the literature [22]. This is the mechanism that has been described for almost 100 years, proving that cancer cells show an increased glycolysis activity, even in the presence of oxygen. Instead of using a more efficient metabolic pathway based on oxidation, malignant tumor cells utilize the conversion of glucose into lactic acid. This allows them to obtain the energy stored in ATP much faster [23,24].

As can be seen from Figure 2, the nitrogen isotopic composition of the tumor is lighter (Δ = δ15Nremote − δ15Ntumor > 0) than the healthy tissue, which is in agreement with literature reports indicating elevations in the anabolism-to-catabolism ratio of the cancer metabolism [17,18,19]. Among the main causes of metabolic changes is the dysregulation of the glutamine metabolism. Endometrial cancer is characterized by the increased uptake of glutamine from the microenvironment [25]. Cancer cells use glutamine, which uses three carbon atoms and two nitrogen atoms, for several metabolic processes. These primarily include the synthesis of purines and pyrimidines [26], amino acids [27], and tricarboxylic acids required for energy homeostasis [28,29].

One of the many mechanisms for regulating the nitrogen metabolism in malignant tumor cells includes the c-Myc-dependent pathway. C-Myc is a gene that encodes transcription factors. Its expression has been shown to increase already at physiological concentrations of glutamine and, through complex metabolic pathways, induces the expression of genes encoding glycolytic enzymes-hexokinase 2 and lactate dehydrogenase [30].

In addition, when glutamine is deficient in the tumor microenvironment, it is possible to replenish this deficit. Glutamine synthetase catalyzes the reaction to convert glutamate and ammonia into glutamine [31,32]. The effect of this process leads to an increase in the availability of glutamine required for nucleic acid synthesis. In scientific experiments, it was further shown that the excessive activity of glutaminase 1 (GLS-1), which catalyzes the reaction of converting glutamine to glutamate and ammonia, stimulates the proliferation of healthy endothelial cells [33]. In cells that have undergone neoplastic transformation, GLS-1 is overexpressed to meet the demand for glutamate and ammonia. The role of ammonia in the growth of cancer cells has also been documented. Ammonia participates in the synthesis of purines and pyrimidines, but its increased concentration inhibits the proliferation of malignant tumor cells [34,35].

Although the glutamine metabolism is the best-studied metabolic pathway in the context of the nitrogen metabolism in cancer cells, metabolic pathways dependent on aspartate and arginine are also currently under investigation [36]. The microenvironment around malignant tumor cells can also affect the nitrogen metabolism through complex regulatory signals. This leads to further changes in the nitrogen distribution within the organ affected by the tumor transformation. The complexity of the nitrogen metabolism in cancer cells and the balance of different metabolic pathways may lead to different isotopic fractionations and, in fact, we have observed opposite Δ-values in the case of cancerous kidney cells (to be published).

A dependence similar to this observed for the nitrogen isotopic composition is also found for the carbon isotopic composition, which also has reported precedence [19], although, in other studies, a higher content of C in tumor tissue [13] compared to healthy tissue has been found and interpreted as an indication of the increased metabolism and cellular respiration of the tumor [37]. It should be noted that, due to the large number of carbon atoms in organic material, it is hard to interpret changes in the carbon isotopic composition, since the EA-IRMS measurements only lead to an averaged value.

According to Warburg’s theory, glucose is used as the main source of carbon for anabolic processes for dynamic cell proliferation [38]. The excess of carbon is diverted to multiple metabolic pathways and used to produce nucleotides, lipids, and proteins. One known metabolic pathway is the synthesis of the amino acid serine by phosphoglycerate dehydrogenase (PHGDH) [39]. In addition to ATP, dynamically proliferating cancer cells also need reducing substances. In this case, the substrate for NADPH production is also organic glucose. In the metabolic pathways described, glucose, with the participation of pentose phosphate, allows for de novo lipid synthesis via reductive biosynthesis [40]. Sources in the literature indicate the existence of an enhanced Warburg effect in endometrial cancer cells [41,42].

Endometrial cancer is, in the vast majority of cases, closely associated with certain risk factors. Metabolic diseases, including hypertension, insulin resistance, obesity, and type 2 diabetes, are often important starting factors for the development of this malignancy [43,44,45]. Facing metabolic syndrome (MetS) in epithelial cells, many mutations occur at the level of mitochondrial DNA, leading to the dysfunction of mitochondrial respiratory chains and oxygen phosphorylation [46]. The discussed Warburg effect also enables the delivery of substrates for angiogenesis in endometrial cancer cells. The products of glycolysis, primarily lactic acid, stimulate the secretion of vascular epithelial growth factor (VEGF) [47,48]. Enhanced angiogenesis, accelerated cell proliferation, and increased tumor mass, as well as several metabolic changes, lead to a vicious cycle mechanism. Currently, many scientific and clinical experiments are being conducted to intervene in tumor glycolysis pathways and induce specific metabolic changes to break the vicious cycle mechanism in terms of the malignant tumor cell metabolism.

Sulfur also plays an important role in the cancer cell metabolism, although we found only a small amount of it (see Figure 3) [9]. In the processes of cellular proliferation, protein synthesis occurs using sulfur-rich amino acids, cysteine, and methionine. The precursor for their formation is the amino acid homocysteine [49]. Obesity (one of the main risk factors for endometrial cancer) is associated with elevated plasma levels of methionine and cysteine [50,51].

Methionine derivatives affect the following:

- Mitochondrial translation initiation [52].
- The synthesis of spermine, spermidine, and putrescine polyamines, which stabilize the structure of nucleic acids and membrane ion channels [53].

Cysteine derivatives play the following role:

- They are a substrate for the synthesis of many compounds that play a role in the metabolism of malignant tumor cells;
- They modify mitochondrial tRNA [54];
- They regulate osmotic processes and play an important antioxidant function for the homeostasis of the tumor microenvironment [55];
- They regulate the glucose and lipid metabolism;
- They affect the processes of oxidative phosphorylation and protein sulfurization;
- They have a positive effect on the processes of angiogenesis, tumor growth, and tumor cell migration [56,57];
- They play a role in the metabolism of malignant tumor cells. In healthy cells, they show strong antioxidant properties and maintain cellular homeostasis [58]. However, it has been shown that, under conditions of severe oxidative stress, elevated glutathione levels contribute to the accelerated progression of malignant tumors and resistance to chemotherapeutic treatment [59,60].

Sulfur is also essential for the synthesis of many other chemical compounds, such as thiols, sulfones, and sulfonotransferases, which play a role in numerous metabolic and antioxidant processes in healthy tissue cells and cancer cells [49,61,62].

We now focus our further discussion on the possible application of the observed differences for diagnostic and prognostic purposes. For the diagnostic purposes, in particular, the noninvasive, the purpose of the relation should be established between the tissue isotopic composition and the serum composition. However, an important issue to consider is that the isotopic composition is closely related to the diet. To overcome the variations in the isotopic composition due to the diet, we have considered the relationship between the serum isotopic composition and the difference between healthy and tumor tissues. This approach should eliminate the dependence on the diet, which may then be considered a common background for both serum and tissue samples. Figure 3 show the results obtained for all three of the studied elements. As can be seen, no correlation can be found in the cases of all studied isotopic compositions. This supports previously reported observations and indicates that the bulk isotopic composition of serum, and that of sulfur in particular, is not a biomarker of endometrial cancer.

To investigate if the obtained results could have any prognostic value, we studied the dependence of the obtained differences and the function of cancer malignancy. For this purpose, the results were grouped according to the following grading: G1, G2, and G3. Unfortunately, no systematic dependence (clustering of the results in groups) has been observed, as illustrated in Figure 3, indicating that the observed changes in the C, N, and S isotopic compositions have no prognostic potentials.

In the studies of kidney cancers, we noticed a significant difference in the total carbon-to-nitrogen ratios ([C]/[N]) between remote and cancerous tissues (to be published). We have, therefore, analyzed these ratios in the present studies. However, the mean values calculated for series B and K, as well as the serum samples, were essentially the same.

## 5. Conclusions

The presented results confirm that cancerous and healthy tissues are characterized by different isotopic compositions of stable isotopes due to different metabolic pathways. They are detectable at the natural abundance, making their measurements potentially diagnostic or prognostic biomarkers. However, no correlation of these differences was manifested in the serum, wherein they would have constituted desired noninvasive biomarkers. Differences in the nitrogen isotopic composition of healthy and cancerous tissues are now well documented, but no correlation with the corresponding isotopic composition of the serum has been thus far reported. In the case of the carbon isotopic composition, there are indications that it might be a useful biomarker. However, the large number of carbon atoms in bio-organic molecules results in isotopic dilution, making the changes hard to interpret. The alleviation of this problem may come from the emerging new techniques, such as isotope ratio Orbitrap, which allows for a position-specific isotopic analysis, which, in the future, might enable us to take advantage of isotopic fractionations in a much broader way [63,64].

Understanding the mechanisms that occur in cancer tissue is important not only in terms of genetics or epigenetics. The isotope analysis of elements not only allows us to understand the changes that occur in cancer, but also creates new possibilities for both diagnostics and treatment.

## 6. Limitations

Our study had some limitations. The first was the small study group, which consisted of only 32 patients. There was also an uneven distribution of patients in the different histopathological groups: G1 consisted of 15 patients, G2 consisted of 12 patients, while G3 consisted of only 5 patients.

Another limitation was the control group, in which the average age was 48.8 years (36–64), while, in the study group with endometrial cancer, it was 66.9 years (46–86). These differences result from the fact that endometrial cancer occurs mainly in older women, in the postmenopausal age. On the other hand, other diseases, e.g., uterine fibroids, most often affect younger women.

## Data Availability

The raw data supporting the conclusions of this article will be made available by the authors on request.
